# Enhancing Patient Safety Through an Integrated Internet of Things Patient Care System: Large Quasi-Experimental Study on Fall Prevention

**DOI:** 10.2196/58380

**Published:** 2024-10-03

**Authors:** Ming-Huan Wen, Po-Yin Chen, Shirling Lin, Ching-Wen Lien, Sheng-Hsiang Tu, Ching-Yi Chueh, Ying-Fang Wu, Kelvin Tan Cheng Kian, Yeh-Liang Hsu, Dorothy Bai

**Affiliations:** 1 Department of Nursing Taipei Veterans General Hospital Taipei Taiwan; 2 School of Nursing National Yang Ming Chiao Tung University Taipei Taiwan; 3 Department of Physical Therapy and Assistive Technology National Yang Ming Chiao Tung University Taipei Taiwan; 4 S R Nathan School of Human Development Singapore University of Social Sciences Singapore Singapore; 5 Gerontechnology Research Center Yuan Ze University Taoyuan Taiwan; 6 School of Gerontology and Long-Term Care College of Nursing Taipei Medical University Taipei Taiwan

**Keywords:** patient safety, falls, fall prevention, fall risk, sensors, Internet of Things, bed-exit alert, motion-sensing mattress system, care quality, quality improvement, ubiquitous health, mHealth

## Abstract

**Background:**

The challenge of preventing in-patient falls remains one of the most critical concerns in health care.

**Objective:**

This study aims to investigate the effect of an integrated Internet of Things (IoT) smart patient care system on fall prevention.

**Methods:**

A quasi-experimental study design is used. The smart patient care system is an integrated IoT system combining a motion-sensing mattress for bed-exit detection, specifying different types of patient calls, integrating a health care staff scheduling system, and allowing health care staff to receive and respond to alarms via mobile devices. Unadjusted and adjusted logistic regression models were used to investigate the relationship between the use of the IoT system and bedside falls compared with a traditional patient care system.

**Results:**

In total, 1300 patients were recruited from a medical center in Taiwan. The IoT patient care system detected an average of 13.5 potential falls per day without any false alarms, whereas the traditional system issued about 11 bed-exit alarms daily, with approximately 4 being false, effectively identifying 7 potential falls. The bedside fall incidence during hospitalization was 1.2% (n=8) in the traditional patient care system ward and 0.1% (n=1) in the smart ward. We found that the likelihood of bedside falls in wards with the IoT system was reduced by 88% (odds ratio 0.12, 95% CI 0.01-0.97; *P*=.047).

**Conclusions:**

The integrated IoT smart patient care system might prevent falls by assisting health care staff with efficient and resilient responses to bed-exit detection. Future product development and research are recommended to introduce IoT into patient care systems combining bed-exit alerts to prevent inpatient falls and address challenges in patient safety.

## Introduction

The challenge of preventing in-patient falls remains one of the most critical concerns in health care. It was reported that the fall rate ranged from approximately 2 to 14 per 1000 patient days depending on the hospital setting and patient characteristics [1-3]. Approximately 15%-50% of patients who fall sustain fall-related injuries, including significant injuries (eg, fractures and lacerations), with approximately 4%-6% leading to severe injuries, which can result in activity restrictions, comorbidities, and even death [2]. In addition, falling is often followed by a prolonged length of stay, which may lead to extra costs and a higher likelihood of patients’ discharge to long-term institutions [4,5]. An additional 11.5 days of length of stay was estimated to occur after a fall [4], with an average cost increase of 61% [6]. It was estimated that medical costs related to both fatal and nonfatal falls in the United States were approximately US $50.0 billion [7]. The US Centers for Medicare and Medicaid Services advised that falls during hospitalization that can reasonably be prevented using evidence-based care should never occur and stopped paying for such preventable conditions since 2008 [3,8].

Various factors are linked to falls, encompassing intrinsic factors such as a history of falls, deficits in gait or balance or vision, chronic diseases, and medication, and extrinsic factors such as inadequate grab bars in bathrooms or toilets, insufficient lighting, bed height, poorly maintained floor surfaces, and incorrect use of assistive devices [9]. Numerous programs to prevent falls have been implemented and studied, encompassing fall risk assessments, patient and family education, technical interventions, and evaluations following a fall [10,11]. The use of technological interventions, such as call buttons, bedside rails, bed-exit detection systems, and environment redesign, among high-risk groups for falling is becoming increasingly common [12,13]. The effectiveness of these technical intervention programs in individual studies varies, and a pooled estimate of multiple studies concluded that there was no statistically significant effect [13-16].

Bed-exit detection systems have become increasingly used technological solutions for inpatient fall prevention [12,15]. Bed-related activities of inpatients were considered and reported to be an essential factor in the clinic, with growing concerns about issues such as a lack of human resources, physical restraints, and pressure injury reduction [17,18]. Bed-exit detection systems include various forms, such as motion-sensing mattresses or pad systems, infrared systems, cameras, and wearable devices [12,14,16,19]. Despite the fact that these solutions are commonly used in clinical practice to prevent falls, the evidence for their effectiveness is insufficient and inconclusive [20-24]. Two studies found that results were positive [20,22], other studies found no significant result [21,24], and 1 study found that there was no effect after controlling for confounders [23]. In addition to the inconclusive findings of the effect of alerting technologies on fall prevention, false alarms remain a barrier to their application in clinical practice [25,26].

This study aims to examine the effect of an innovative smart patient care system (SPCS) on inpatient fall prevention. The specific objective was to compare the inpatient fall incidences between patients who were admitted to wards that used a traditional patient care system (TPCS) and those admitted to wards that used an SPCS.

## Methods

### Recruitment

We used a quasi-experimental study design to investigate relationships between an Internet of Things (IoT) patient call system and inpatient fall occurrence. Participants were recruited from 4 wards of the Gastroenterology and Hepatology Department of a medical center in Taiwan over a 12-month period. The department’s acquisition of the smart system, prompted by rising fall rates and a high incidence of false alarms with the traditional system, provided a timely opportunity for a convenient sample. This enabled us to investigate the impact of an IoT patient care system on patient safety. With the institutional review board (IRB) waiving the requirement for informed consent, we recruited all patients admitted to this department during the study period, resulting in a total of 1300 participants. The sample size was determined prior to recruitment, with a significance level set at 5% and a desired power level of 80%. Recruitment concluded upon reaching this predetermined sample size. The inclusion criteria were patients administrated to 4 wards of the Gastroenterology and Hepatology Department who were aged over 20 years and who spoke Chinese or Taiwanese. Exclusion criteria were patients who were bedridden during hospitalization, and those with medical conditions that would preclude the use of the SPCS, for example, patients using an air cushion bed.

### Fall Risk and Injury Assessments

Apart from the different patient care systems, participants in all 4 wards received standard care following departmental health care guidelines. To compare the characteristics of participants from the TPCS and SPCS wards, we measured their age, sex, and length of stay in the ward together with a fall risk assessment. In the Gastroenterology and Hepatology Department, health care staff were required to complete the fall risk assessment test for all patients using comprehensive fall risk scores within 24 hours after admission. The comprehensive fall risk scores measured were based on scores of the St Thomas’s Risk Assessment Tool in Falling Elderly Inpatients (STRATIFY) plus the polypharmacy situation. The STRATIFY instrument is a simple fall risk-assessment tool consisting of 5 items: history of falling, patient agitation, visual impairment affecting everyday function, need for frequent toileting, and transfer ability and mobility [27]. Although STRATIFY has been widely used to assess fall risks since its development, its ability to predict falls among older adults is unsatisfactory. A multicenter study that recruited 2568 patients showed that although the STRATIFY showed satisfactory sensitivity (≥84%) and a high negative predictive value (≥99%) for the total sample, the sensitivity (approximately 52%-69%) and specificity (approximately 55%-71%) for patients admitted to geriatric wards and for patients aged 75 years and older were moderate to low [28]. For the comprehensive fall risk scores of the site of this study, the polypharmacy status (yes or no, with “yes” defined as using 4 or more different medicines when recruited) was added, which was shown to be a significant predictor in previous studies [29]. At the study site, a patient was considered to have polypharmacy if he or she used 4 or more different medicines at the same time. The total comprehensive fall risk score corresponded to the sum of the STRATIFY score and the polypharmacy status (yes=1 and no=0) and ranged from 0 to 6. A score of 3 or greater was accepted as a high risk of falls.

According to the Taiwan Quality Indicator Project followed by most hospitals in Taiwan, the severity classification of fall injuries includes 3 grades: grade 1 (minor injuries such as scratches, abrasions, small skin tears, or cuts that only require observation or minimal treatment); grade 2 (injuries such as sprains, large or deep cuts, or small abrasions that require medical or nursing intervention such as ice, bandaging, stitching, or splinting); and grade 3 (injuries such as fractures, loss of consciousness, or changes in body posture that require medical intervention and consultation, and may significantly affect the patient’s condition or lead to an extended hospital stay).

### Instruments

The TPCS is the current patient care system used in most wards at this medical center and has been used for over 30 years. The SPCS was set up in 1 ward of the Gastroenterology and Hepatology Department, and all relevant health care staff in that ward were subsequently trained to use this system. Both the TPCS and SPCS were used as systems for receiving and managing patient calls or bed-exit alerts. Details of the differences between the TPCS and SPCS were described in a previously published article [30]. The SPCS, which is an integrated IoT patient care system, comprises a motion-sensing mattress for bed-exit monitoring, a patient call button, the health care staff’s mobile devices, and the health care staff scheduling system. Integrated patient care provided by the SPCS aims to minimize hospital-acquired falls and reduce the care burden on health care staff.

**Figure 1:**
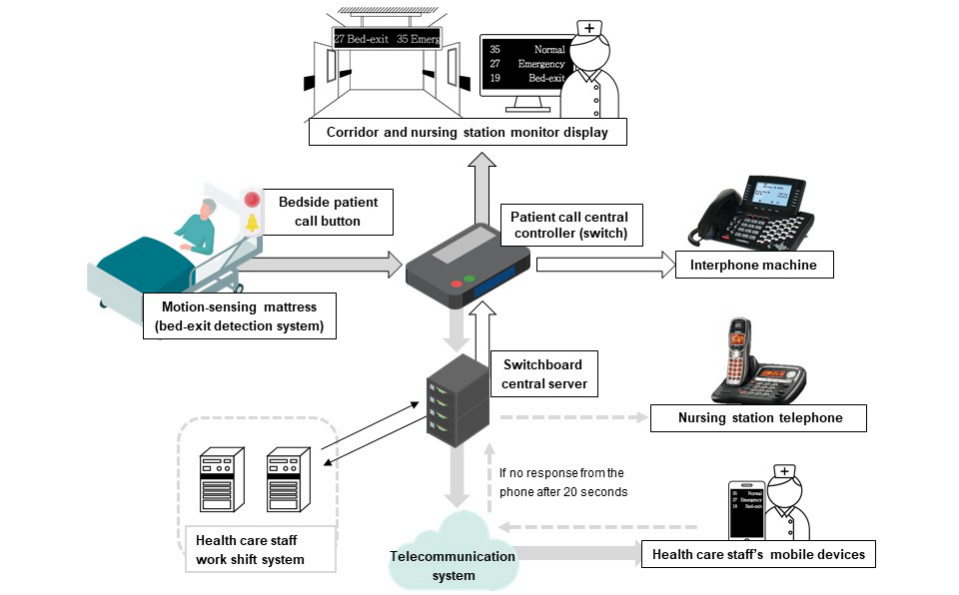
The signal transduction pathway of the smart patient care system.

The signal transduction pathway of the SPCS is shown in Figure 1. In this pathway, there are several advantages of the SPCS compared to the TPCS. First, in the SPCS, the type of patient calls such as normal, emergency, and bed-exit, can be recognized, and a signal is sent to health care staff’s mobile devices to help them receive the alert without the limitation of location. Second, the information exchange system in SPCS is connected to the scheduling system in the hospital so that only the health care staff on duty and responsible for a certain patient will receive such alarms. Third, this system also allows immediate communication between health care staff and patients. When the health care staff receive an alarm, they can directly speak to the patient, when necessary, by phone, and the patient can hear them by the speaker near the call button or bed. For example, if a health care staff member receives a bed-exit alert but cannot immediately provide assistance, he or she can answer the alarm by phone and ask the patient not to leave the bed until he or she comes to assist.

**Figure 2:**
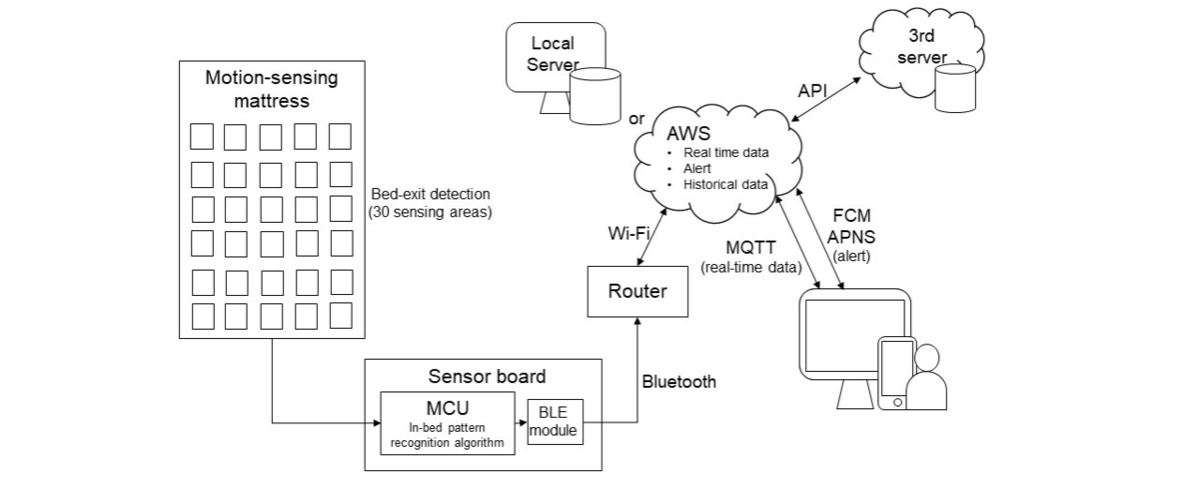
The information technology structure of the bed-exit detection system in the smart patient care system. API: application programming interface; APNS: Apple Push Notification service; AWS: Amazon Web Services; BLE: Bluetooth Low Energy; FCM: Firebase Cloud Messaging; MCU: multiprocessor communications unit; MQTT: message queuing telemetry transport.

The bed-exit alert is used for patients at high risk of falling. All call alarms can be canceled at a distance by monitors or phones, except for the bed-exit alert which needs to be canceled by pressing a bedside button to ensure that actual assistance has arrived. In wards with TPCSs, a movable sensing mat was used for the bed-exit alert which was activated in the same way as patient calls. Again, only the bed number is shown, and health care staff are unable to tell whether it is a bed alarm or a patient call. In wards with SPCSs, a motion-sensing mattress plays an important role in notifying health care staff of a patient’s real-time in-bed positions. The IT structure of the fall-preventing motion-sensing mattress system in SPCS consists of 30 sensing areas, a sensor board, a wireless router, and a server (Figure 2). Sensing data are collected when users lie or sit in bed via the 30 sensing areas of membrane switches and then sent to the sensor board which contains a multiprocessor communications unit and a Bluetooth Low Energy module.

The motion-sensing mattress uses a multilayer perceptron neural network, a machine learning method, to generate the algorithm to identify a user’s position, including on-bed, bed-edge, and off-bed. The final algorithm is loaded onto the sensor board, and position pattern results are sent to the wireless router by the Bluetooth Low Energy module and then sent to the database by Wi-Fi. In this system, Amazon Web Services or sometimes a local server is normally used as the server, and a third server can also access the data via an application programming interface with permission. On one hand, the server sends position pattern results to health care staff’s mobile devices or computers for real-time event display by message queuing telemetry transport and sends the real-time position alert by Firebase Cloud Messaging or Apple Push Notification service. On the other hand, the server is used as the database for further analysis of historical data or user or device information input and settings.

### Statistical Analysis

Descriptive analyses were used to describe the characteristics of participants using the mean, frequency, and percentage depending on the variables. Chi-square tests, Fisher exact tests, and 2-tailed *t* tests were also conducted to compare participant characteristics in the TPCS and SPCS groups. Fisher exact tests were used to compare whether there was a significant difference in the occurrence of falling between the 2 groups. In addition, unadjusted and adjusted logistic regression models were used to investigate the effect of the SPCS on fall prevention during hospitalization. The confounding variables included in the regression model were age, sex, length of hospital stay, and fall risk. The Hosmer-Lemeshow goodness-of-fit test was used to assess the adequacy of the logistic models, and the variance inflation factor was used to assess multicollinearity [31,32]. All data analyses were performed using the statistical software package Stata statistical software (version 16; StataCorp). A nominal significance level of .05 and a power of 80% were used throughout the analysis.

### Ethical Considerations

The study procedures were reviewed and approved by the IRB of Taipei Veterans General Hospital, Taipei, Taiwan (2017-07-017B). The IRB waived the requirement for informed consent. Additionally, all data collected for this study were anonymized to ensure the privacy and confidentiality of participants. No compensation was provided to participants for their involvement in the study.

## Results

**Figure 3:**
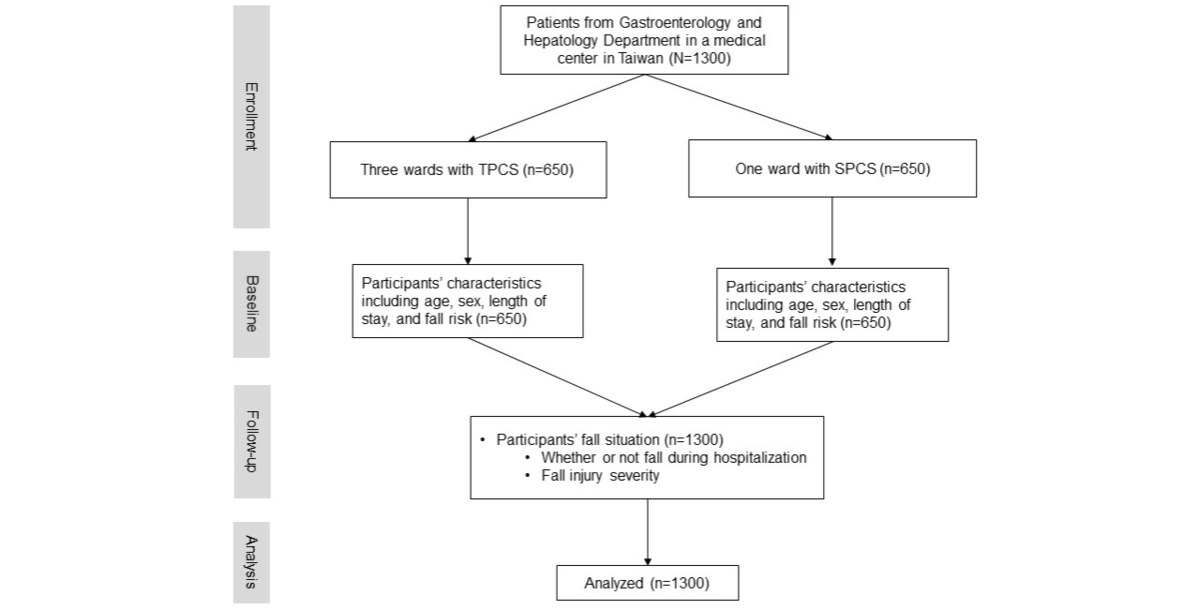
Flow diagram of participants. SPCS: smart patient care system; TPCS: traditional patient care system.

In total, 1300 patients (from 4 wards of the Gastroenterology and Hepatology Department) were recruited, followed up, and analyzed in this study (Figure 3). Participants’ demographic characteristics and the incidence of bedside falls were compared between the TPCS and SPCS wards (Table 1). The average age was 67.02 years in the TPCS ward and 67.81 years in the SPCS ward. There was no significant difference in the age distribution between the TPCS and SPCS wards but with some difference in sex. In the TPCS ward, approximately 466 (71.7%) participants were male, compared to about 305 (46.9%) participants in the SPCS ward. Among all participants, 733 (56.4%) had a stay of less than 1 week, while 246 (18.7%) stayed for more than 2 weeks, with no significant differences between the 2 groups. Fall risks of the 2 groups were found to significantly differ, with 320 participants (49.2%) recognized as being at some level of risk in the TPCS ward, whereas in the SPCS ward, 365 participants (56.2%) were considered at risk. The total bedside fall incidence during hospitalization was 0.7% (n=9), with 8 (1.2%) in the TPCS ward and 1 (0.1%) in the SPCS ward. The participants in the SPCS group who fell had no clinical injury. Among the 8 participants who fell in the TPCS ward, 3 had no clinical injury, 3 had a grade 1 injury, 1 had a grade 2 injury, and 1 had a grade 3 injury. Results from Fisher exact test suggested a significant difference in the fall incidences between the TPCS and SPCS wards.

**Table 1:** Characteristics of participants (N=1300).

			Total		TPCS^a^ (n=650)		SPCS^b^ (n=650)		*P* value	
Age (years), mean (SD)			66.93 (14.97)		67.02 (14.92)		67.81 (15.01)		.46	
**Sex, n (%)**										<.001
	Male	771 (59.3)		466 (71.7)		305 (46.9)				
	Female	529 (40.7)		184 (28.3)		345 (53.1)				
**Length of stay (days), n (%)**										.06
	<7	733 (56.4)		348 (53.6)		385 (59.2)				
	8-14	324 (24.9)		166 (25.5)		158 (24.3)				
	>15	243 (18.7)		136 (20.9)		107 (16.5)				
**Fall risk scores, n (%)**										.02
	0	615 (47.3)		330 (50.8)		285 (43.8)				
	1	150 (11.6)		79 (12.1)		71 (10.9)				
	2	354 (27.2)		165 (25.4)		189 (29.1)				
	>3	181 (13.9)		76 (11.7)		105 (16.2)				
**Fall, n (%)**										.04
	No	1291 (99.3)		642 (98.8)		649 (99.9)				
	Yes	9 (0.7)		8 (1.2)		1 (0.1)				

^a^TPCS: traditional patient care system.

^b^SPCS: smart patient care system.

In the SPCS ward, the IoT patient care system equipped with a 3-stage bed-exit alarm system detected an average of 13.5 potential falls per day, with no false alarms recorded throughout the study period. In contrast, the TPCS wards issued approximately 11 alarms daily, of which roughly 4 were false, effectively identifying 7 potential falls using the traditional system. The IoT system enhanced operational efficiency by transmitting alarm types directly to health care staff’s mobile devices and monitors located in the corridors. This capability reduced the proportion of alarms addressed at the nursing station to 10.9% in the SPCS wards, with the majority being managed proximally to the health care staff’s locations at the time of alert. This system design significantly reduced workflow interruptions compared to the TPCS wards, where approximately 28.2% of the alarms required health care staff to communicate with patients via the nursing station telephone or return to verify alarm types, thus disrupting other duties.

We used unadjusted and adjusted logistic regression models to investigate the relationship of the use of the SPCS with falling (Table 2). We found that the likelihood of falling in the SPCS ward was reduced by 88% (odds ratio [OR] 0.12, 95% CI 0.02-0.99; *P*=.049) compared to the TPCS ward. After adjusting for confounding variables, this result remained significant and showed that the SPCS was associated with an 88% reduced risk of falling (OR 0.12, 95% CI 0.01-0.97; *P*=.047). In addition, with one more score of the comprehensive fall risk scores, the likelihood of actual falling was significantly increased 2.88 times (OR 2.88, 95% CI 1.28-6.48; *P*=.01) even after adjusting for confounding variables. Results of the Hosmer-Lemeshow goodness-of-fit tests for the adjusted logistic model indicated that it was a good fit for the data. Variance inflation factor values also indicated a low degree of multicollinearity.

**Table 2:** Unadjusted and adjusted OR^a^ of fall according to the wards and participants’ characteristics (N=1300).

			Unadjusted			Adjusted					
			OR (95% CI)		*P* value	OR (95% CI)		*P* value			
**Group**											
	TPCS^b^			1.00 (—^c^)	—		1.00 (—)		—		
	SPCS^d^		0.12 (0.02-0.99)		.049	0.12 (0.01-0.97)		.047			
	Fall risk scores^e^		2.96 (1.39-6.33)		.005	2.88 (1.28-6.48)		.01			
Age			1.05 (1.00-1.11)		.048	1.03 (0.97-1.08)		.33			
**Sex**											
	Male		1.00 (—)		—	1.00 (—)		—			
	Female		0.41 (0.09-2.00)		.27	0.55 (0.11-2.77)		.47			
**Length of stay (days)**											
		<7	1.00 (—)		—	1.00 (—)		—			
		8-14	2.27 (0.46-11.33)		.32	1.35 (0.26-7.03)		.72			
		>15	3.04 (0.61-15.17)		.18	1.37 (0.26-7.18)		.71			

^a^OR: odds ratio.

^b^TPCS: traditional patient care system.

^c^—: not applicable/not available.

^d^SPCS: smart patient care system.

^e^The total comprehensive fall risk score corresponds to the sum of St Thomas's Risk Assessment Tool in Falling Elderly Inpatients score and the polypharmacy status (yes=1, no=0) and ranges between 0 and 6. A score of 3 points or higher predicts a higher likelihood of falling.

## Discussion

### Principal Findings

This study investigated an integrated IoT patient care system’s effect on inpatient fall prevention with a large prospective cohort. The SPCS is an IoT patient care system that consists of bed-exit monitoring by a motion-sensing mattress, patient call button, health care staff’s mobile devices, and health care staff scheduling system for integrated patient care aimed at reducing hospital-acquired falls and health care staff’s care burdens. The advantages of the SPCS were that it specifies the source of a signal as general, emergency, and bed-exit alerts; the health care staff can immediately receive an alarm and communicate with the patient via a mobile device. The 3-stage bed-exit alerts of the motion-sensing mattress help the health care staff determine a patient’s intention of leaving the bed and offering help if necessary. We found that the incidence of hospital-acquired falls was significantly reduced by 88% in the ward using the SPCS compared to that using the TPCS.

It is common for falls to occur around beds in both hospital and household settings [1-3,33,34]. Falls also frequently occur between 5 PM and 7 AM in rooms with lower staffing levels [19]. Similarly, a recent report from the Department of Health in Taiwan showed that of all inpatient falls, approximately 78.9% occurred between 6 PM and 6 AM, and around one-quarter of falls were related to getting in or out of bed [35]. Increasing the human resources or workload of the health care staff is less likely to prevent bed-related falls given the consideration that human resources are most likely to be short in most conditions [36]. An alternative and rapidly emerging strategy for fall prevention is bed-exit detection systems that provide a possible solution by detecting and alerting patients and health care staff by providing alarms when a patient is at high risk of falling when trying to leave their bed unassisted [14,37,38].

The bed-exit detection system is commonly used in clinical practice, and studies even observed a significant increase in their use after the US Centers for Medicare and Medicaid Services stopped paying for preventable hospital-acquired falls and shifted the burden to hospitals [39]. Bed-exit detection systems can be categorized as ambient, wearable types and various forms of mattress or pad systems, wearable devices, and video systems using infrared sensors or cameras. Although wearable devices are usually portable and easy to use, they are usually designed to detect a fall event instead of predicting it and sending an alert before the fall happens, which limits their application in fall prevention and makes them less effective in preventing falls [40-42]. Although some researchers have been trying to design body-worn devices that detect the rising attempts, patients’ adherence to wearing such devices remains a barrier [22]. Video monitoring is another kind of bed-exit detection system to predict the occurrence of falls, the acceptance of which by the public, however, is generally lower than 50% in various settings, let alone considering potential legal issues of such a system [43].

With less adherence and acceptance problems, mattress or pad systems provide a proper solution to reduce falls by sending the health care staff alerts when at-risk patients attempt to leave their beds without assistance (nonrestrictive). Bed-exit alerts usually use pressure-sensitive sensors to collect pressure signals of a patient’s weight, and alarms are triggered when a patient tries to get up from the bed and the pressure on the sensor is relieved [37,44]. Despite their widespread use, the evidence for the effectiveness of bed-exit alerts in preventing falls is insufficient and inconsistent [16,20,45]. Two before-and-after studies showed that bed-exit alerts were associated with reduced fall events by 18% and 54% [23]. One cluster randomized trial also showed their significant effectiveness [46]. In contrast, many other studies did not find a significant reduction in fall rates when using bed-exit alerts [20,21,24,42,46]. A clinical trial study that recruited 70 participants from a geriatric ward showed that a bed-exit alert using pressure-sensitive sensors had no significant effect on fall prevention [24]. A more recent clustered randomized trial that recruited 27,672 inpatients from 16 nursing units in an urban community hospital in the United States suggested no significant effect on hospital-acquired falls or the use of physical restraints [21]. Another clustered randomized study showed no effect [42].

In this study, we observed a significant association between the SPCS and a reduction in hospital-acquired falls. Apart from the fall rate being low and it being hard to detect a significant effect [21,24,44], there are several plausible explanations for why the SPCS was significantly effective in fall prevention. First, it is common for pressure-sensitive sensors to send alarm signals after a patient has already exited the bed, which leads to limited time for health care staff to come and assist so that a fall is more likely to occur [43]. Moreover, some sensors are designed to delay the alert for about 2 seconds after detecting the pressure change to reduce false alarms, which, however, could also be a barrier to providing timely assistance by health care staff [44]. The system in this study adopted an algorithm that allows the mattress to send signals in 3 stages: a patient sits on the bed, sits on the edge of the bed, and exits the bed. The health care staff can choose whether to receive the early-stage notifications according to a patient’s comprehensive situation of fall risks and family assistance. This graduated sequence of bed-exit notifications provides health care staff additional time to respond to the alerts. A pilot study that investigated the effect of a medical IoT system for fall prevention using similar technology to provide early notifications when a patient attempted to leave the bed showed no bed-related falls during 234 patient days [47].

One of the principal challenges encountered in implementing this study was designing the IoT system that could be integrated into health care staff's daily routines. The goal was to enhance patient safety through the use of this system, rather than imposing additional burdens that might lead to its eventual abandonment despite its innovative potential. In addition to sending alerts to a dashboard at the nursing station or a monitor in the corridor, the SPCS can also notify health care staff via an app on their mobile devices so that they can receive the alerts wherever and whenever it is necessary. The system is connected to the internet at the hospital as well as the health care staff scheduling system so that the health care staff will only receive an alert when they are on duty. Similarly, a study in an acute-care setting showed that the use of an alarm system with a portable beeper carried by nursing staff was associated with a reduction in fall incidence [23]. Although there is a clinical strategy to move patients at high risk of falling to units near the nursing station for better monitoring, on one hand, this could increase the workload of administration arrangements, and on the other hand, there is a lack of evidence of its effectiveness. With a portable device that can receive alerts, patients’ locations would not influence the nursing staff’s response times [47]. With the IoT system, the portable and immediate reception of an alert would help health care staff decrease the response time and provide help in a timely manner whenever necessary without the patient being placed near the nursing station.

Furthermore, it was shown that health care staff’s experiences with bed-exit alarm systems influenced their effectiveness. The SPCS has an immediate communication function through which health care staff can talk to patients on a mobile device once they receive an alert. Health care staff have multiple responsibilities and need to decide the priority of a new task when it comes in. Without enough information on the source and emergency level of a patient call, health care staff are more likely to assume it is nonurgent and choose to handle it after they finish what they are doing, which may lead to a fall when immediate assistance is truly needed [48]. For example, when health care staff receive a bed-exit alert, but they cannot drop what they are doing, it will help if they can talk to the patient at high risk of falling to instruct them to wait until they can come to assist or maybe ask other colleagues to help handle it. Otherwise, a patient call signal can more easily be ignored especially when the purpose of the signal is not specific [49]. With the immediate communication function of the SPCS, bed exits are no longer an extra burden but are well integrated into the health care process.

Last but not least, false alarms are commonly reported with existing bed-exit alert systems. It was shown that false alarms accounted for greater than 80% of all bed-exit alerts, leading to alarm fatigue, which can increase the health care staff’s work burden, lower their willingness to use the system, and ultimately result in patients falling [25,50,51]. Excessive false alarms may lead to alarm fatigue in which health care staff will occasionally ignore an alarm. A multicenter study showed that over 70% of monitor alarms were false alarms, and only 5.9% of all alarms received a response from health care staff [52]. In addition, false alarms may frequently disrupt patients’ sleep and negatively influence their recovery [53]. Our previous results showed that it took around 2.5 times longer for health care staff to respond to bed-exit alerts in TPCS wards than that in SPCS wards [30]. The high predictive positive value of the SPCS significantly improved health care staff’s willingness to use the system, which may be another reason that the SPCS had a positive effect on fall prevention [54].

### Strengths and Limitations

This was a large prospective cohort study that examined the association between an IoT patient care system and inpatient falls. Despite this, it has some limitations. First, the study was not population-based and used a convenience sample, which may introduce selection bias and limit the generalizability of the findings to a broader population. Additionally, the sample was drawn from a single site, further restricting the applicability of the results to other settings. Generalization of the findings to the entire inpatient population would be limited. The proportion of female participants was higher in the SPCS group than in the TPCS group. The existing evidence shows that sex is not statistically associated with inpatient falls [55,56]. In addition, health care staff were aware that the effect of SPCS on falls was being studied, and it is possible that they were more vigilant when the bed alarms occurred according to the Hawthorne effect [57]. However, this study lasted for around 1 year, and it is likely that the active responses to patient calls including bed-exit alerts were integrated into their daily practice, which was one of the purposes of the system. Another potential limitation of this study was the use of logistic regression to analyze fall events, which are relatively rare in our dataset. Logistic regression models can sometimes underestimate the likelihood of rare events occurring. This underestimation may affect the accuracy of the predicted probabilities; therefore, our results should be interpreted with caution.

### Conclusions

Bed-exit detection systems have been increasingly used around the world for inpatient fall prevention. They are also commonly combined with patient care systems to help notify health care staff of bed-exit alerts. Unfortunately, many of the current bed-exit detection and patient care systems are connected in a traditional signal transduction way instead of a more efficient method using IoT techniques. The SPCS introduced in this study integrated a motion-sensing mattress with bed-exit alerts as well as a patient call button, health care staff’s mobile devices, and a health care staff scheduling system. The design of this IoT patient care system allows health care staff to immediately receive necessary bed-exits alerts by mobile devices without location limitations, which will help them provide timely assistance for at-risk patients or easily cope with an alarm while they are in the middle of other duties. As a result, the efficient and resilient responses to bed-exit detection are expected to help with fall prevention in hospitals. Future research should focus on refining IoT integration techniques within health care systems, enhancing predictive analytics for early detection, expanding interoperability standards, and assessing the longitudinal impact of IoT-enabled systems on patient safety and care efficiency, particularly in reducing hospital-acquired falls.

## Abbreviations

IoT: Internet of Things
IRB: institutional review board
OR: odds ratio
SPCS: smart patient care system
STRATIFY: St Thomas's Risk Assessment Tool in Falling Elderly Inpatients
TPCS: traditional patient care system

## Data Availability

The datasets generated during and/or analyzed during this study are available from the corresponding author on reasonable request.
